# Mineralization of the *Callorhinchus* Vertebral Column (Holocephali; Chondrichthyes)

**DOI:** 10.3389/fgene.2020.571694

**Published:** 2020-11-26

**Authors:** Jacob B. Pears, Zerina Johanson, Kate Trinajstic, Mason N. Dean, Catherine A. Boisvert

**Affiliations:** ^1^School of Molecular and Life Sciences, Curtin University, Perth, WA, Australia; ^2^Department of Earth Sciences, Natural History Museum, London, United Kingdom; ^3^Department of Biomaterials, Max Planck Institute of Colloids and Interfaces, Potsdam, Germany

**Keywords:** Holocephali, *Callorhinchus*, tesserae, mineralization, evolution, stem group Holocephali

## Abstract

Members of the Chondrichthyes (Elasmobranchii and Holocephali) are distinguished by their largely cartilaginous endoskeletons, which comprise an uncalcified core overlain by a mineralized layer; in the Elasmobranchii (sharks, skates, rays) most of this mineralization takes the form of calcified polygonal tiles known as tesserae. In recent years, these skeletal tissues have been described in ever increasing detail in sharks and rays, but those of Holocephali (chimaeroids) have been less well-studied, with conflicting accounts as to whether or not tesserae are present. During embryonic ontogeny in holocephalans, cervical vertebrae fuse to form a structure called the synarcual. The synarcual mineralizes early and progressively, anteroposteriorly and dorsoventrally, and therefore presents a good skeletal structure in which to observe mineralized tissues in this group. Here, we describe the development and mineralization of the synarcual in an adult and stage 36 elephant shark embryo (*Callorhinchus milii*). Small, discrete, but irregular blocks of cortical mineralization are present in stage 36, similar to what has been described recently in embryos of other chimaeroid taxa such as *Hydrolagus*, while in *Callorhinchus* adults, the blocks of mineralization are more irregular, but remain small. This differs from fossil members of the holocephalan crown group (*Edaphodon*), as well as from stem group holocephalans (e.g., Symmorida, *Helodus*, Iniopterygiformes), where tesserae are notably larger than in *Callorhinchus* and show similarities to elasmobranch tesserae, for example with respect to polygonal shape.

## Introduction

During ontogeny, most vertebrate skeletons are initially composed predominantly of hyaline cartilage and largely replaced by bone via endochondral ossification (Hall, 1975, 2005). In contrast, chondrichthyans, including elasmobranchs (sharks, skates, rays, and relatives) and holocephalans (chimaeroids) do not develop osseous skeletons, having secondarily lost the ability to produce endoskeletal bone (Coates et al., 1998; Dean and Summers, 2006; Ryll et al., 2014; Debiais-Thibaud, 2019; Brazeau et al., 2020). Instead, the chondrichthyan endoskeleton remains primarily composed of hyaline-like cartilage, with elasmobranchs developing a comparatively thin outer layer of cortical mineralization over most of their skeleton during ontogeny (Hall, 2005; Egerbacher et al., 2006; Dean et al., 2009, 2015; Seidel et al., 2016, 2019b; Atake et al., 2019; Debiais-Thibaud, 2019). This mineralized tissue begins as small separated islets near the cartilage surface, which gradually grow via mineral accretion to fill the intervening spaces, eventually forming a cortex of abutting polygonal tiles called tesserae (Dean and Summers, 2006; Dean et al., 2009, 2015; Seidel et al., 2016, 2019b). These tiles cover the uncalcified cartilage core and are themselves overlain by a distal fibrous perichondrium (Dean and Summers, 2006; Dean et al., 2009, 2015). This mosaic of uncalcified cartilage, tesserae and perichondrium is called tessellated cartilage and comprises most of the cranial and postcranial skeleton (Kemp and Westrin, 1979; Dean and Summers, 2006; Seidel et al., 2016, 2017a).

Tessellated cartilage is therefore a major component of the skeleton and is currently believed to be a synapomorphy for the entire chondrichthyan group (e.g., Maisey et al., 2019, 2020, but see comments therein regarding morphological and histological disparity in stem-chondrichthyans). Contemporary examination of extant chondrichthyan mineralized skeletons and their tissues, however, have almost exclusively focused on sharks (Kemp and Westrin, 1979; Peignoux-Deville et al., 1982; Clement, 1986, 1992; Bordat, 1987, 1988; Egerbacher et al., 2006; Eames et al., 2007; Enault et al., 2016) and rays (Dean et al., 2009; Claeson, 2011; Seidel et al., 2016, 2017a,b; Criswell et al., 2017a,b). In contrast, mineralized skeletal tissues of extant chimaeroids (Holocephali) have been largely ignored, since the descriptions of vertebral development and morphology in the late nineteenth to mid-twentieth centuries (Hasse, 1879; Schauinsland, 1903; Dean, 1906); fossil holocephalans have faced similar neglect (but see Moy-Thomas, 1936; Patterson, 1965; Maisey, 2013). This has led to contradictory descriptions of chimaeroid tissues (Lund and Grogan, 1997; Grogan and Lund, 2004; Pradel et al., 2009), prompting calls for more research (Eames et al., 2007; Dean et al., 2015; Enault et al., 2016). Notably, recent examination of chimaeroid mineralized skeletal tissues identified tesseral structures in the vertebral column (synarcual) and Meckel’s cartilage of *Chimaera* and *Hydrolagus* (both Family Chimaeridae; Finarelli and Coates, 2014; Debiais-Thibaud, 2019; Seidel et al., 2019a, 2020) and in the fin skeleton of *Callorhinchus* (Family Callorhinchidae; Maisey et al., 2020), seemingly refuting the view that extant chimaeroids lack tessellated cartilage.

In order to address this controversy, and determine whether tessellated cartilage is indeed a shared character among cartilaginous fishes, we performed a correlated, multi-technique examination of mineralization in the skeletal tissue of *Callorhinchus*, focusing on the synarcual of the elephant shark (*Callorhinchus milii*). The synarcual is a fused element in the anterior vertebral column (Claeson, 2011; Johanson et al., 2013, 2015, 2019; VanBuren and Evans, 2017) and is one of the better anatomical structures for mineralized tissue characterization, being formed early in development and also mineralizing early (Johanson et al., 2015, 2019). Whilst no developmental series of synarcual mineralization in *C. milii* has yet been published, two observations from the only study of its development (Johanson et al., 2015) suggest its spatial pattern. The formation of the cartilages that comprise the synarcual of *C. milii* occurs anteroposteriorly. Micro-CT analysis of an adult sample, from the same study, suggested an anterior-to-posterior/dorsal-to-ventral mineralization front. Accordingly, we assume that antero-dorsal mineralized tissues in *C. milii* are more advanced developmentally than ventro-posterior mineralized tissues, which appears to be reflected in the histological data examined here. We report the presence of a layer of mineralization in the *Callorhinchus* embryo, maintained in adults, comprising small, irregularly shaped units, but lacking many of the characteristics of tesserae in the elasmobranchs. To provide phylogenetic context we also examined mineralized tissues in fossil members of the Callorhinchidae (*Edaphodon*; Nelson et al., 2006), as well as stem-group holocephalan taxa (e.g., *Cladoselache*, *Cobelodus*, *Helodus*, Iniopterygiformes; Coates et al., 2017, 2018; Dearden et al., 2019; Frey et al., 2019). The tesserae in these stem-group holocephalans are larger than in *Callorhinchus*, and more similar in shape to polygonal elasmobranch tesserae. Thus, the evolution of skeletal mineralization in Chondrichthyes may have involved a progressive reduction of mineralization in the Holocephali, relative to the elasmobranchs.

## Materials and Methods

### Histological Sections of *Callorhinchus milii* Synarcual

To gain insight into the development of mineralized tissues, stained slides of the synarcual from a sectioned embryo of an elephant shark (*Callorhinchus millii*; section thickness ∼30 μm; Life Sciences Department, Natural History Museum, London) were examined by light microscopy using an Olympus BX51 compound microscope and Olympus DP70 camera and management software. These slides were prepared sometime during the 1980s and no information aside from the species was recorded with the slides. They are presumed to be stained with haematoxylin and eosin. The animal is estimated to represent stage 36 [near hatching, based on the calculated size of the individual (110–135 mm; Didier et al., 1998)]. This developmental stage is ideal to study mineralization as it is small enough to section but mature enough to show mineralization. As noted, given that holocephalan synarcuals are known to mineralize first anterodorsally and that mineralization subsequently progresses in a posteroventral fashion (Johanson et al., 2015), this provides ontogenetic information on how mineralization develops, in one individual. Location of hard tissue was confirmed by micro-CT scanning of the sections.

### Adult *Callorhinchus milii*

Two adult females of *C. milii* were captured by rod and reel from Western Port Bay, Victoria, Australia (Permits: RP1000, RP 1003, and RP1112) with the authorization and direction of the Monash University Animal Ethics Committee (Permit: MAS-ARMI-2010-01) and kept according to established husbandry methods (Boisvert et al., 2015). These specimens died in captivity and were frozen.

### Scanning Electron Microscopy

**FIGURE 7:**
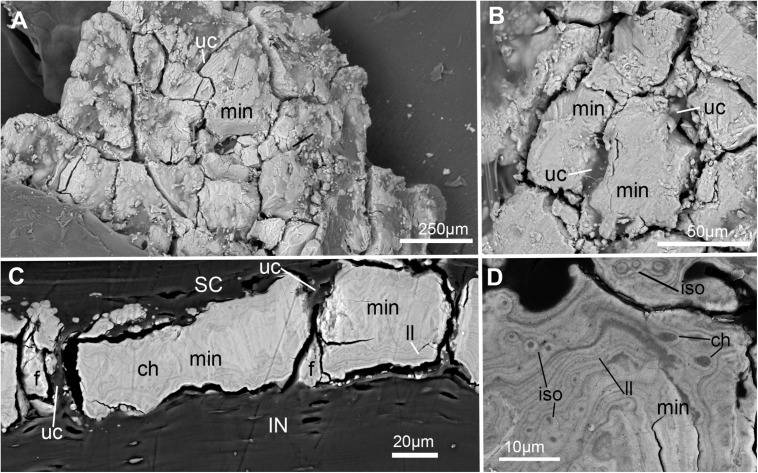
SEM images of mineralization from the synarcual (anterior fused vertebrae) of an adult *Callorhinchus milii* (Holocephali; Callorhinchidae). **(A)** Overview of tesselated mineralization from a planar perspective; **(B)** close up of mineralization from a planar perspective; **(C)** mineralization from a transverse perspective; **(D)** close up of mineralization surface in transverse perspective; Note brightness and contrast of **(C,D)** have been altered to more clearly visualize morphology. Abbreviations: As in previous Figures, also f, fragments ll, Liesegang lines.

The synarcual of one of these adult *C. milii* specimens was dissected out and either small layers of mineralized tissue or cross sections of the vertebrae were collected. Samples were macerated in a trypsin solution (0.25 g Trypsin Sigma T-7409 Type II-S from porcine pancreas in 100 mL 10%PBS) and warmed in a 38°C water bath. Samples were extracted from the solution every hour to remove macerated flesh and fascia using scalpels, needles and forceps. This was repeated until sufficient flesh had been removed to observe the mineralized surface. To prevent distortion, samples were placed between Teflon blocks before being air-dried until firm. Cross sections were embedded in a Struers CitoVac using Struers EpoFix Resin and EpoFix Hardener mixed in a 50:6 weight ratio and polished using a Struers Tegramin-30. All samples were given a 3 nm conductive coating of pure platinum using a Cressington 208HR sputter coater. Samples were imaged using a TESCAN MIRA3 XMU variable pressure field emission scanning electron microscope (VP-FESEM) using backscatter mode (voltage: 15 kv; working distance: 6–15 mm; Tescan Mira3 VP-FESEM instrumentation, John de Laeter Centre, Curtin University). The synarcual from the remaining adult specimen (Johanson et al., 2015; Figure 7) was dissected out, defleshed by immersion in 36°C water and removal of muscle and fascia with needles and forceps, and imaged using a FEI Quanta 650 FEG SEM in secondary electron mode (voltage: 10 kv; working distance: 14.7 mm). Through this method mineralized tissues can be easily distinguished from soft tissue through differences in backscatter signal.

### Macrophotography, CT-Scanning

Five fossil holocephalans from the Earth Sciences Department, NHM (NHMUK PV P) were chosen to represent extinct taxa, phylogenetically important with respect to the Callorhinchidae and crown-group holocephalans (Coates et al., 2017, 2018; Frey et al., 2019). These comprised: *Cladoselache* (NHMUK PV P.9285), *Cobelodus* (NHMUK PV P.62281a), *Sibirhynchus* (NHMUK PV P.62316b), *Edaphodon* (NHMUK PV P.10343), and *Helodus* (NHMUK PV P.8212). One specimen preserving mineralized cartilage was chosen from each taxon, and photographed using a Canon EOS 600D camera, EOS Utility. Five to ten images of each specimen were taken at different focal depths and the resultant image stack imported into Helicon Focus (v. 6.8.0) to create images with high depth of focus. These specimens were also photographed using a Zeiss Axio Zoom microscope with camera to provide closeup images; tesseral width was determined using the measurement function in the Zen Pro 2 software accompanying the Axio Zoom microscope (Supplementary Info Table 1).

The second adult *Callorhinichus milii* synarcual was CT-scanned (Johanson et al., 2015) using an X-Tek HMX ST CT scanner (Image and Analysis Centre, NHM; *kv* = 165; μA = 175; no filter applied; 3142 projections; resolution = 36.9μm), and rendered using the programs Drishti^1^ and Avizo^2^. Subsequently, the synarcual was air-dried and photographed using the Zeiss Axio Zoom microscope to illustrate tesseral shape.

## Results

### Histology

#### General Morphology

The axial skeleton of chondrichthyans typically includes a series of cartilages dorsal and ventral to the notochord, and in the elasmobranchs, centra associated with the notochord (e.g., Dean, 1895; Gadow and Abbott, 1895; Goodrich, 1930; Compagno, 1977; Criswell et al., 2017b). Mineralization of the axial skeleton takes a variety of forms, recently summarized by Debiais-Thibaud (2019), with the dorsal and ventral cartilages (i.e., neural and haemal arches) of most species, as well as the outer centrum, composed of tessellated cartilage (Dean and Summers, 2006; Dean et al., 2009; Criswell et al., 2017b; Johanson et al., 2019). Most of the spool-shaped vertebral centrum comprises areolar mineralization, with substantial variation in patterns of mineralization between elasmobranch species (Ridewood, 1921; Dean and Summers, 2006; Porter et al., 2007). Holocephalans also possess dorsal and ventral cartilages (e.g., Dean, 1895; Johanson et al., 2013, 2015), but centra do not develop (Gadow and Abbott, 1895; Goodrich, 1909). Instead, the notochord is surrounded by a fibrous chordal sheath, which contains many calcified rings, except in the Callorhinchidae, where these rings are absent (Goodrich, 1909; Patterson, 1965; Didier, 1995). Holocephalans, unlike many elasmobranchs, possess a synarcual, which is the focus of the following description.

**FIGURE 1:**
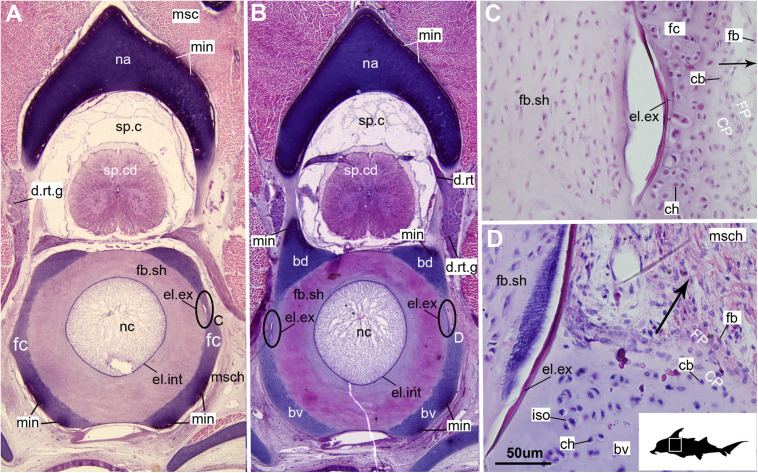
Histological sections through the synarcual (anterior fused vertebrae) of a stage 36 embryo of *Callorhinchus milii* (Holocephali; Callorhinchidae). **(A,B)** Section showing neural arch surrounding the spinal cord and basidorsal and basiventral arches surrounding the notochord. **(C)** closeup of region indicated in **(A)**; **(D)** closeup of region indicated in **(B)**. Black s in **(C,D)** indicate direction of appositional growth of the basiventral cartilage. bd, basidoral; bv, basiventral; cb, chondroblast; ch, chondrocyte; CP, perichondrium including chondroblast cells; d.rt, dorsal root of the spinal nerve; d.rt.g, dorsal root ganglion of the spinal nerve; el. ex, elastica externa; el.int, elastica interna; fb, fibroblasts; fb.sh, fibrous sheath surrounding the notochord; fc, fused cartilage; FP, perichondrium including fibroblast cells; iso, isogenous group of chondrocytes; min, mineralization; msc, musculature; msch, mesenchymal cells; na, neural arch; nc, notochord; sp.c, spinal cavity; sp.cd, spinal cord. Black silhouette of *C. millii* indicates approximate region shown in the figure.

In the *Callorhinchus* embryo examined (stage 36), several tissue layers concentrically surround the notochord. Most proximal is a thin basophilic membrane, the elastic interna, adherent to the outside of the notochord (Figures 1A–D, nc, el.int). Distal to this membrane is a thick (∼665 μm) fibrous sheath (Figures 1A,B, fb.sh), which is largely composed of spindle shaped cells (Figures 1C,D). Abutting the sheath dorsally and ventrally are separate bilateral pairs of cartilages, the basidorsals and basiventrals, respectively (Figures 1B,D, bv, bd). Immediately dorsal to the sheath is the spinal cavity, containing the spinal cord, which is surrounded ventrolaterally by the basidorsal cartilages and dorsally by the neural arch cartilage (Figure 1B, sp.c, sp.cd, bd, na). Spinal nerves are also visible in section, with the dorsal root exiting the neural tube toward the dorsal root ganglion situated lateral to the vertebral column (Figures 1A,B, d.rt, d.rt.g). The hyaline cartilages associated with the vertebral column—the neural arch, basidorsals and basiventrals— fuse anteriorly to form the synarcual, which surrounds the majority of the fibrous sheath and spinal cavity, while maintaining foramina for the dorsal root (Figure 1A).

**FIGURE 2:**
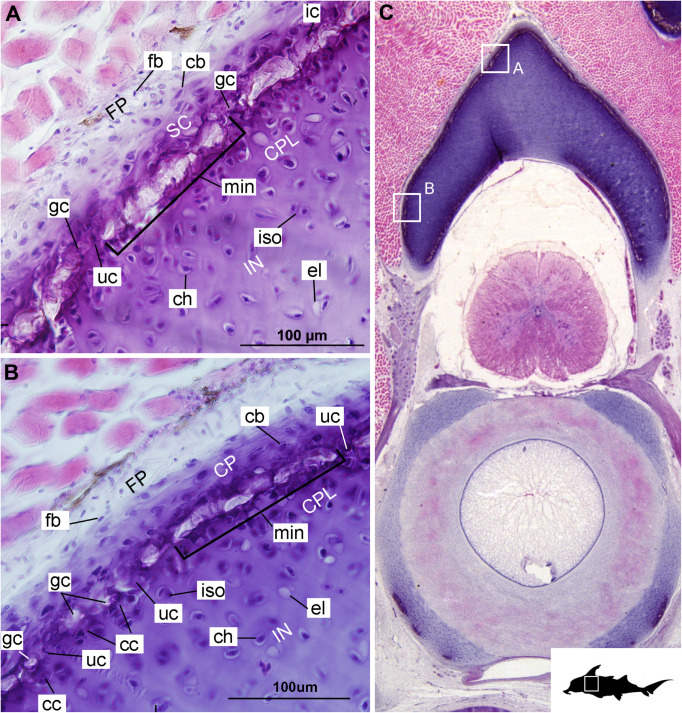
Histological section through the anterior synarcual (anterior fused vertebrae) of a stage 36 embryo of *Callorhinchus milii* (Holocephali; Callorhinchidae). **(A,B)** Closeups showing perichondrium, cartilage, and mineralization in the neural arch; **(C)** overview of section with locations of closeup views indicated by white squares. Abbreviations: As in Figure 1, also cc, clustered chondrocytes CPL, chondrocyte proliferative layer; el, empty chondrocyte lacunae; gc, calcification globule; ic, chondrocyte that is being engulfed or has been incorporated; uc, uncalcified cartilage; IN, internal cartilage; SC, supratesseral/mineral cartilage. Black silhouette of *C. millii* indicates approximate region shown in the figure.

**FIGURE 3:**
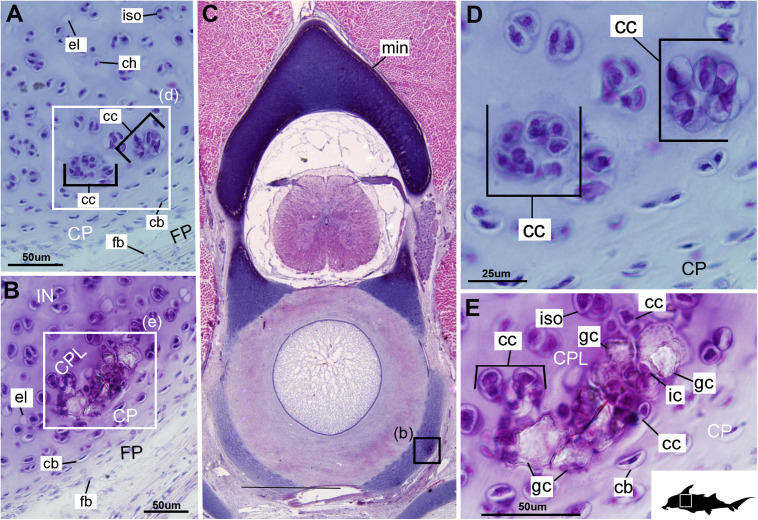
Histological sections through the posterior synarcual (anterior fused vertebrae) of a stage 36 embryo of *Callorhinchus milii* (Holocephali; Callorhinchidae). **(A,B)** Closeups showing initial mineralization in a basiventral and clustered chondrocytes in the same location in the preceding section; **(C)** overview with location of closeup view of initial mineralization indicated by a black square; **(D,E)** close ups of **(A,B)** locations indicated by white squares. Abbreviations: As in previous figures, also dm, developing mineralization. Black silhouette of *C. millii* indicates approximate region shown in the figure.

In these histological slides, areas of mineralization, verified via CT imaging, are limited to the superficial regions of the vertebral column-associated cartilages (Figures 1, 2, min). These mineralized tissues are bordered externally by a fibrous perichondrium and a thin, cell-rich layer of cartilage (Figures 2, 3, FP, SC), similar to the supratesseral cartilage intervening between tesserae and perichondrium in the stingray *Urobatis halleri* (Seidel et al., 2017a).

#### Cellular Aspects

Cells within the neural arch, basidorsal and basiventral cartilages can be categorized with respect to morphology, and distribution within the cartilage. Chondrocytes located proximally inside the cartilage (Figures 2A,B, 3A, IN, ch) are generally similar in terms of cell morphology and density: ≥200 μm from the periphery, cells are sparsely distributed within the cartilage matrix, with most being ovoid in shape and located in open circular spaces identified as lacunae (diameter: ∼15 μm). Chondrocytes often occur in pairs (isogenous groups), indicative of recent mitotic activity (Figures 2A,B, 3A, ch; Kheir and Shaw, 2009). This proximal region (≥200 μm from the periphery) of cartilage also contains relatively greater quantities of empty lacunae compared to the more peripheral cartilage (Figures 2A,B, 3A, el). Closer to the periphery, within 100–200 μm of the outer edge, and immediately proximal to mineralized tissue, chondrocytes are clustered within a distinct layer (Figures 2, 3B, CPL) and appear uniformly ovoid. This area displays a greater variation in cell size, as it contains many smaller chondrocytes (diameter: 5–10 μm) and fewer empty lacunae compared to the interior. In addition, this area contains notably more isogenous groups relative to the interior, which may indicate higher rates of chondrocyte proliferation (Figures 2A,B, 3B,E, iso; Kheir and Shaw, 2009). In some regions, mineralization is absent at the periphery; in these areas, cell distribution and morphology are more similar to the interior (Figure 3A).

#### Mineralization

**FIGURE 4:**
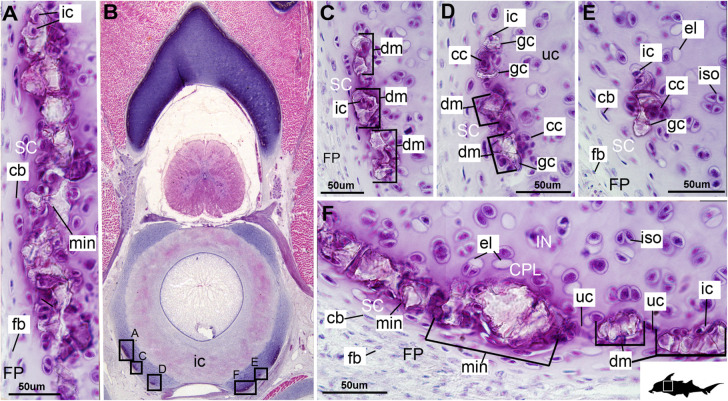
Histological section through the anterior synarcual (anterior fused vertebrae) of a stage 36 embryo of *Callorhinchus milii* (Holocephali; Callorhinchidae). **(A,C–F)** Closeups showing perichondrium, cartilage, and mineralization in basiventrals; **(B)** overview of section through the neural arch with locations of closeup views indicated by black squares. Abbreviations: As in previous figures. Black silhouette of *C. millii* indicates approximate region shown in the figure.

**FIGURE 5:**
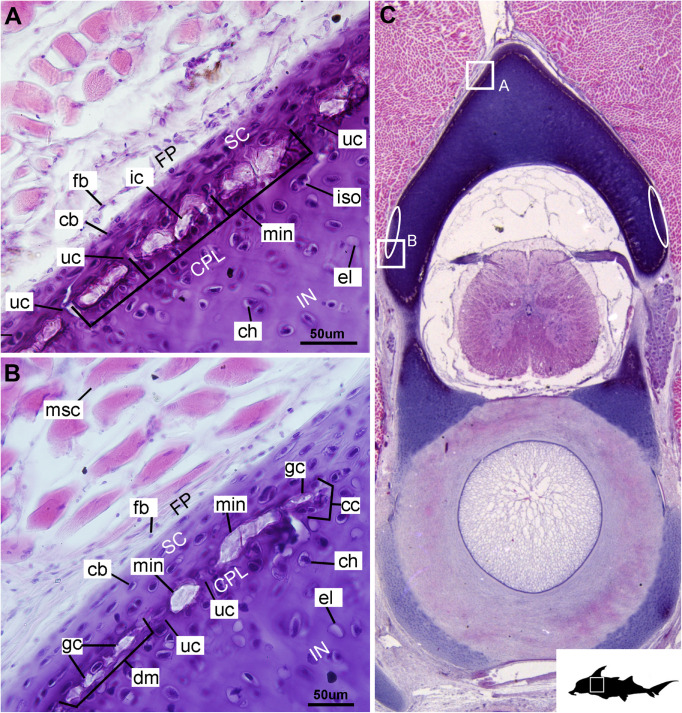
Histological section through the posterior synarcual (anterior fused vertebrae) of a stage 36 embryo of *Callorhinchus milii* (Holocephali; Callorhinchidae). **(A,B)** Closeups showing perichondrium, cartilage, and mineralization in the neural arch; **(C)** overview of section with locations of closeup views indicated by white squares. Abbreviations: As in previous figures, also ellipses indicating areas not yet mineralized. Black silhouette of *C. millii* indicates approximate region shown in the figure.

**FIGURE 6:**
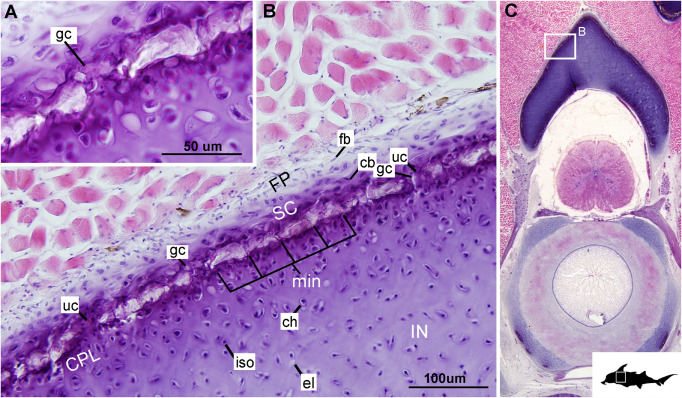
Histological section through the synarcual (anterior fused vertebrae) of a stage 36 embryo of *Callorhinchus milii* (Holocephali; Callorhinchidae). **(A,B)** Closeups showing the potential formation of new mineralization foci between already existing units; **(C)** overview of section through the neural arch with locations of closeup views indicated by white squares. Abbreviations: as in previous figures. Black silhouette of *C. millii* indicates approximate region shown in the figure.

The layer of mineralization varies in its completeness in the vertebral elements, but typically appears discontinuous, broken into individual acellular units; we identify these units as tesserae, although they are not entirely similar to the tesserae of sharks and rays (discussed further below). In the neural arches, more dorsally, the distribution of mineralized tissue is more complete, extending along almost the entire periphery, excluding only the ventro-mesial concave part of the arch (Figures 2C, 4B). Within the basiventrals, mineralized tissue is also found near the periphery, but by comparison to the neural arches, is only patchily distributed (Figures 3, 4), with individual units more variable and irregular in shape (Figures 3B,E, 4, min). In the neural arches, these units are more rectangular and flatter (Figures 2A,B, 5A,B, 6). Nevertheless, mineralized tissues in all vertebral elements lack a regular geometry and any differentiation into inner and outer regions. Additionally, beyond being limited to the cartilage periphery beneath the fibrous perichondrium, these tissues lack obvious patterning, reflecting the lack of a regular geometric shape to the individual units.

The least developed forms of tissue mineralization, which are located in the more posterior vertebrae, take the form of small islands of calcification (≤25 μm width) situated amongst concentrated clusters of chondrocytes beneath the perichondrium (Figures 3A,D, 5B, cc, FP, gc). More developed tesserae in the posterior vertebrae are smaller (50–100 μm) and more regularly separated by regions of unmineralized cartilage (Figures 4F, 5A,B, min, uc), reflecting their earlier developmental stage. Unlike the more developed acellular units in the anterior vertebrae (Figures 2A,B, min) some units in the posterior vertebrae appear to contain vital chondrocytes (Figures 3F, 5A, ic).

### Scanning Electron Microscopy (SEM)

In planar views of the external surface of the synarcual of adult *Callorhinchus milii*, the mineralized layer appears to comprise a tessellated surface of irregular tiles that are separated by (∼5 μm) thin strips of uncalcified cartilage (Figures 7A,B min, uc). These tesserae do not have a uniform shape or size, ranging from 50 to 150 μm in width (Figure 7B and Supplementary Figure 1).

In transverse view, the mineralized tissue forms a single layer of tesserae, tightly arranged units of irregular blocks separated by very thin (<5 μm) strips of uncalcified cartilage (Figure 7C, min, SC, IN, uc). In this perspective, the tesserae are 30–50 μm thick and 30–150 μm wide (Figure 7C). Some cracking of the mineralization during sample preparation is visible, but individual tesserae can be identified by comparing and matching Liesegang lines between adjacent fragments (Figure 7C, ll, f). Liesegang lines are concentric, wave-like patterns of varying mineral density visible in the mineralized tissue, and are particularly prominent near the lateral margins of the mineralized units (Figures 7C,D, ll).

Spheroidal mineralized regions, surrounded by Liesegang lines and approximately the size and shape of chondrocytes also permeate the tesserae (Figure 7D, ch). These are likely calcified (micropetrotic) cells, are variously sized (∼1–5 μm), and appear to be organized in clusters (isogenous groups), suggesting some have either been calcified during mitosis or immediately after mitosis, but before interstitial growth separated the cells in an isogenous groups (Seidel et al., 2016, 2017b, 2019c; Figure 7D, ch, iso).

### Mineralization in Stem Holocephali and Fossil Callorhinchidae

**FIGURE 8:**
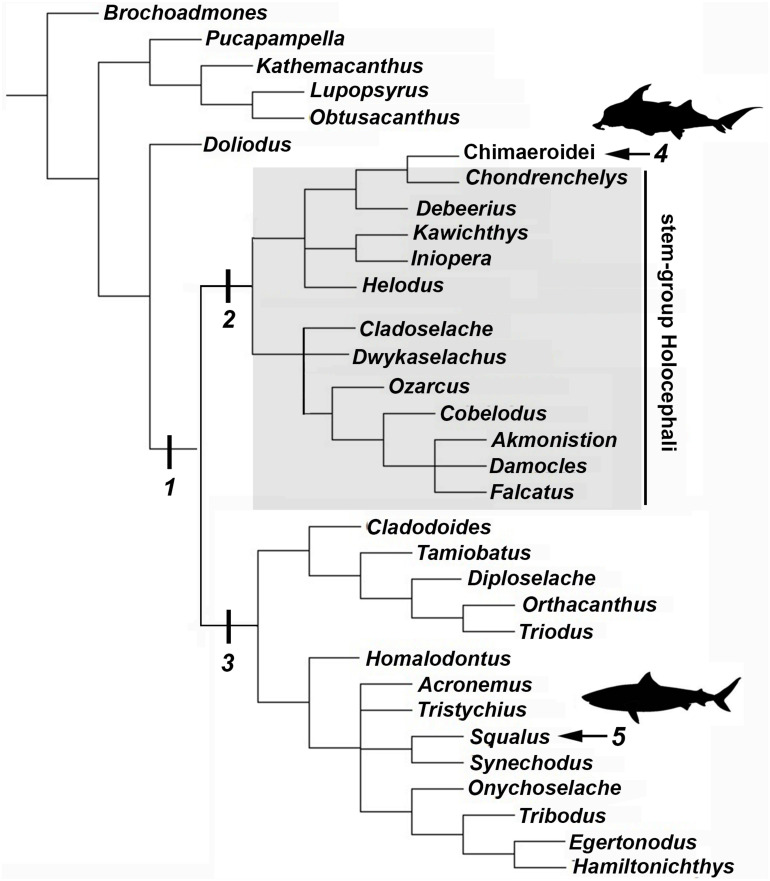
Chondricthyan phylogeny (after Coates et al., 2018). 1, Crown–group Chondrichthyes (Holocephali + Elasmobranchii); 2, Holocephali; 3, Elasmobranchii; 4, Crown–group Holocephali; 5, Crown–group Elasmobranchii.

**FIGURE 9:**
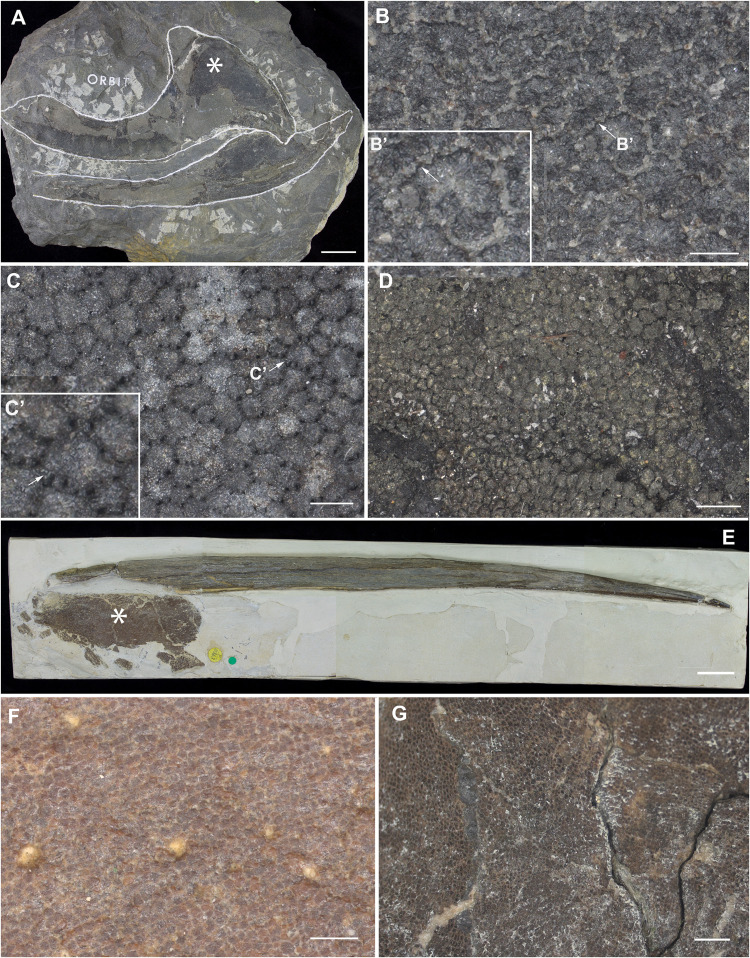
Mineralized cartilage in stem Holocephali and crown group Holocephali (Figure 8). **(A)** NHMUK PV P.9285, *Cladoselache*, stem Holocephali, palatoquadrate, and Meckel’s cartilage; asterisk indicates area shown in **(B)**, anterior to the left; **(B)** polygonal mineralization (tesserae), with irregular margins; **(B’)** closeup of tessera, white arrow indicates contact along the tesseral margin which may represent “spokes” characterizing elasmobranch tesserae; **(C)** NHMUK PV P.62281a, *Cobelodus*, stem Holocephali, cranium, more regular polygonal mineralization (tesserae); **(C’)** closeup of tessera, white arrow indicates “spokes” more similar to those in elasmobranch tesserae; **(D)** NHMUK PV P.62316b, *Sibirhynchus*, stem Holocephali, cranium, polygonal mineralization (tesserae); **(E,F)** NHMUK PV P.10343, *Edaphodon*, Family Callorhinchidae, crown group Holocephali (Figures 8, 2), **(E)** dorsal fin endoskeletal support (with dorsal fin spine, anterior to left), asterisk indicates area shown in **(F)**, anterior to the left; **(F)** closeup showing polygonal mineralization (tesserae); **(G)** NHMUK PV P.8212, *Helodus*, cranium, polygonal mineralization. See Supplementary Info Table 1 for tessera sizes in these taxa.

**FIGURE 10:**
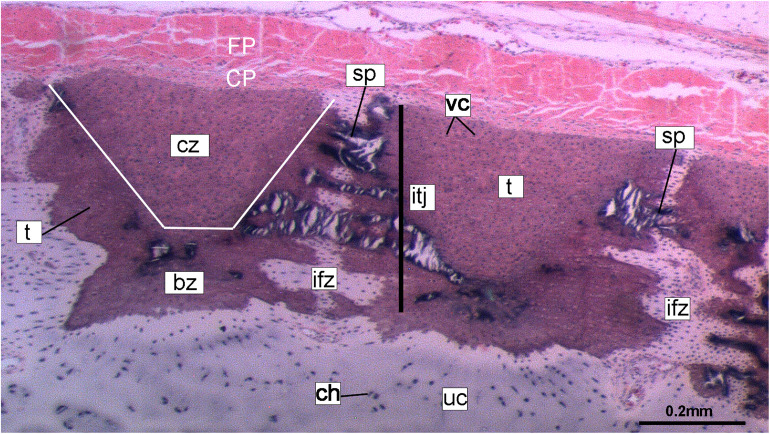
Histological section of tessellated cartilage of a batoid ray (*Raja*). Abbreviations: as in previous Figures also bz, body zone; CP, chondrogenic perichondrium cz, cap zone; ifz, intertesseral fibrous zone; itj, intertesseral joint; FP, fibrous perichondrium; sp., spoke; t, tesserae; vc, vital chondrocytes.

Following recent phylogenetic reviews (Coates et al., 2017, 2018; Dearden et al., 2019; Frey et al., 2019), several taxa that were previously resolved as stem group chondrichthyans (basal to the clade Elasmobranchii + Holocephali; Pradel et al., 2011) are now resolved as stem holocephalans. These taxa join more crownward stem holocephalans including the Iniopterygiformes (Zangerl and Case, 1973), *Helodus* (Moy-Thomas, 1936), *Kawichthys*, *Debeerius*, and *Chondrenchelys*, the latter being the sister taxon to the crown group Holocephali (chimaeroids) (Figure 8). Tessellated calcified cartilage has been variously identified among these stem-group Holocephali: this includes taxa assigned to the Symmoriida, such as *Dwykaselachus* (Coates et al., 2017: extended data Figure 1D), *Ozarcus* (Pradel et al., 2014), *Cladoselache* (“minute granular calcifications,” Dean, 1894; Figures 9A,B), *Akmonistion* (“prismatic calcified cartilage,” Coates and Sequeira, 2001), *Damocles* and *Falcatus* (Lund and Grogan, 1997), also present in *Cobelodus* (Figure 9B). In all these taxa, the tessellated layer is comprised of recognizable polygonal units, although in *Cladoselache*, the edges of the units appear less regular, and were referred to as “zig-zag tesserae” (Maisey et al., 2020). This may represent the presence of mineralized “spokes” extending between the tesserae (Maisey et al., 2020; Figure 9B, arrows): spokes are hypermineralized tissue regions associated with points of contact between elasmobranch tesserae, often represented externally by lobulated extensions along tesseral margins (Seidel et al., 2016; Atake et al., 2019; Jayasankar et al., 2020; Figure 10). Such structural extensions, suggestive of mineralized spokes, are even more clearly present in *Cobelodus* (Figure 9C, arrows).

In the more crownward stem holocephalans, comparable polygonal tesserae are also present, including in *Kawichthys* (“tesserate prismatic calcified cartilage,” Pradel et al., 2011) and the Iniopterygiformes (“calcified cartilage prisms,” Zangerl and Case, 1973), represented by *Sibirhynchus* in Figure 9D. Particularly small tesserae (Supplementary Info Table 1) are present in *Helodus* (“minute tesserae,” Moy-Thomas, 1936; Figure 9G), and *Chondrenchelys* (“tessellated calcified cartilage,” Finarelli and Coates, 2014; Figure 7B). There appears to be more variation in the shape of these polygons, and signs of mineralized spokes are less apparent in these taxa, but this may be due to postmortem distortion. With respect to the fossil taxa assigned to the Callorhinchidae (crown group Holocephali), mineralized tissue units in *Edaphodon* appear to maintain a polygonal shape, compared to the stem holocephalans just described (Figures 9E,F). The width of tesserae in these fossil taxa was measured (Supplementary Info Table 1) for comparison to the size of mineralized units in adult *Callorhinchus* (50–150 μm, as noted above); the tesserae of all fossil taxa were notably larger than in *Callorhinchus*, discussed further below.

## Discussion

### Cartilage Growth and Cellular Aspects

The cartilages in the histological series examined here exhibit tissue and cellular morphologies that suggest mechanisms of growth and cell death were occurring in these tissues. The perichondria of the cartilages, for example, most notably the basidorsals and basiventrals, display a gradient of cell morphology that may explain one of the means by which these cartilaginous tissues grow. From the perichondrium through to the supratesseral cartilage, there is a transition in cellular morphology from fibroblast cells in the perichondrium, differentiating to chondroblasts in the supratesseral cartilage, and then to chondrocytes in the main body of the cartilage, suggesting a potential progressive differentiation of cell type between regions, as described in Genten et al. (2009; Figures 1C,D, 2A,B, CP, FP, fb, cb, ch). This is suggestive of appositional growth, in which newly differentiated chondrocytes deposit matrix at the cartilage margins. Interstitial growth, that is, growth through chondrocyte mitosis and matrix deposition increasing the size of the cartilage element from within, also appears to be occurring as evidenced by the presence of isogenous groups (groups of recently divided chondrocytes, e.g., Figures 1B–D, 2A,B, iso; Hall, 2005; Kheir and Shaw, 2009). Both processes seem to be involved in the formation of the synarcual. In several sections, the basidorsal and basiventral cartilages are still separate (e.g., Figure 1B), but show marginal regions suggestive of appositional and interstitial growth, which continues until these unite to form the synarcual (Figures 1B–D, black arrows suggesting direction of growth). Both modes of growth have similarly been speculated to be involved in the growth and development of the metapterygium in elasmobranchs (Marconi et al., 2020).

Regarding cell death, empty lacunae are found throughout the cartilages examined here, but they appear in greater numbers more proximally (>200 μm from the periphery) (Figures 2A,B, 3A, el). This may indicate either an artifact of sectioning or chondroptosis (chondrocyte apoptosis; Roach et al., 2004), although the latter in vertebrates is normally associated with chondrocyte hypertrophy, which has not been observed in chondrichthyans (Dean et al., 2015; Seidel et al., 2017b; but see Debiais-Thibaud (2019) for a summary of contrary opinions).

### Mineralized Tissue Development

**FIGURE 11:**
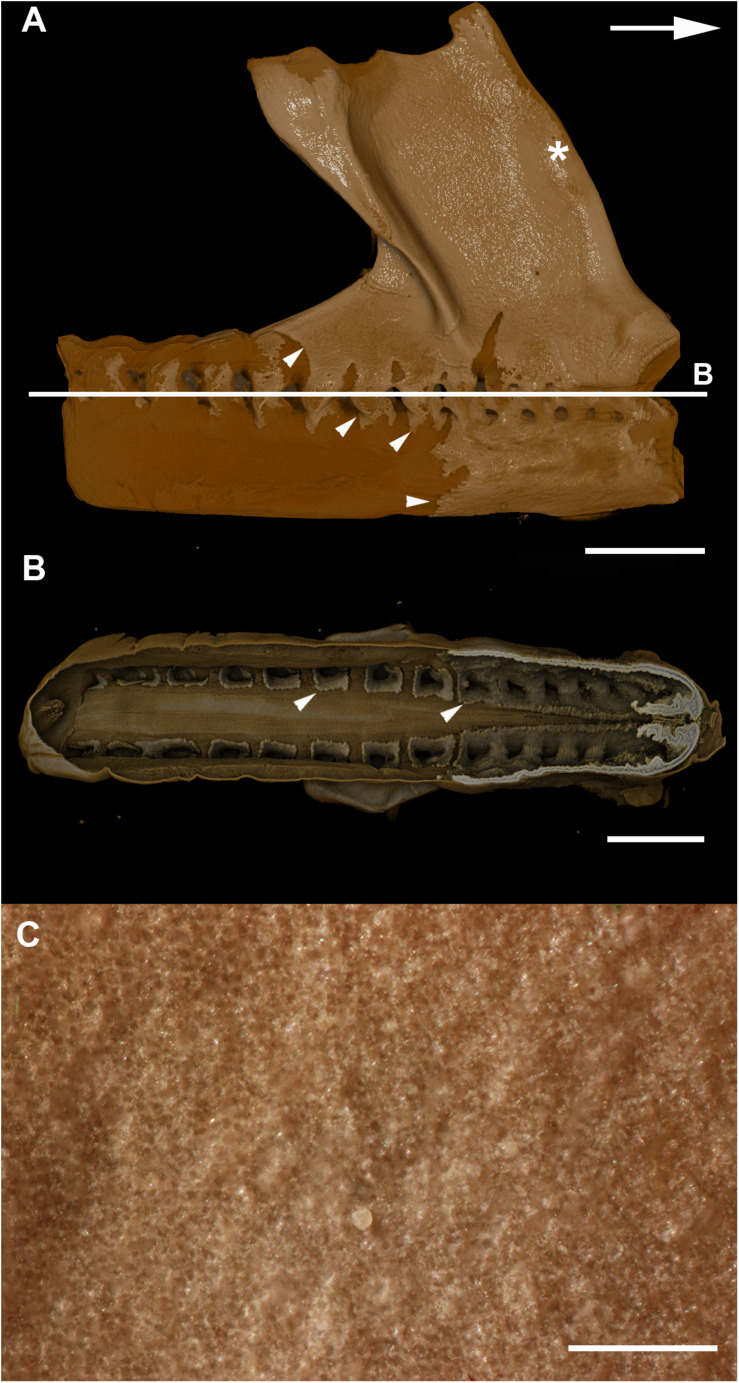
**(A,B)** micro–CT scan of a synarcual from an adult *Callorhinchus milii* (Holocephali; Callorhinchidae). **(A)** synarcual, lateral view; **(B)** coronal section (virtual) through synarcual; **(C)** macrophotograph of lateral synarcual surface showing mineralization with a granular appearance. Figures 11A,B from Johanson et al. (2015). Usage permitted under CC BY licensing.

Currently, the ultrastructure and ontogeny of mineralized endoskeletal tissues of chimaeroids is poorly described, with previous work only providing a broad overview of developmental trajectories, such as observations that mineralization in the vertebral skeleton (in the synarcual) progresses from anterior to posterior and dorsal to ventral, demonstrated by a recognizable mineralization front in a micro-CT scan of a *Callorhinchus milii* adult (Johanson et al., 2015; Figures 11A,B, white arrowheads). The series described above in the stage 36 embryo goes beyond this to capture fine histological detail related to the progression of this mineralization.

The development of mineralized tissue described here for *C. milii* shares some similarities with the development of elasmobranch tesserae (Seidel et al., 2016; Debiais-Thibaud, 2019). Tesserae in elasmobranchs such as the batoid ray *Urobatis halleri* initially develop as patches of globular mineralization interposing within clusters of flattened, subperichondral chondrocytes (at a distance from the perichondrium). These chondrocytes become entombed by the growth of these mineralized inter-chondrocyte septa, apparently by mineral accretion (Dean et al., 2009; Seidel et al., 2016). The morphologies associated with this accretion and entombment process in elasmobranchs (e.g., the size and shape of mineralized septae) are similar to those we observed in *C. milii*, suggesting a similar inception and progression of mineralization (Figures 4C,D, 5A,B). Through development of *U. halleri*, the mineralized septae continue to grow and engulf chondrocytes, eventually forming discrete, but abutting tesserae, which contain vital chondrocytes and closely border the perichondrium (Seidel et al., 2016; also in the batoid *Raja clavata*, Debiais-Thibaud, 2019). In a general sense, elasmobranch tesserae are not dissimilar to some of the developing units of mineralization in *C. milii* (Figure 4F, dm, ic), which also border the perichondrium, grow via calcification of surrounding cartilage matrix and, at least early in development, contain chondrocytes which appear to be vital. Additionally, mineralized tissues in *C. milii* appear to be overlain by a distinct layer of uncalcified cartilage, beneath the perichondrium (Figure 2A, SC). This resembles the thin layer of “supratesseral uncalcified cartilage” intervening between tesserae and perichondrium in elasmobranchs such as *U. halleri* and *Scyliorhinus canicula* (Kemp and Westrin, 1979; Bordat, 1988; Egerbacher et al., 2006; Enault et al., 2015; Seidel et al., 2016, 2017a; Debiais-Thibaud, 2019).

Despite these similarities, however, mineralization in adult *C. milii* appears to result in a distinct form of tessellated calcified cartilage. From a planar perspective, the mineralized tissue comprises a more irregular mosaic of tesserae than typically seen in elasmobranchs, with near-abutting calcified tiles separated by uncalcified cartilage (Figures 7A,B, min, uc; Supplementary Figure 1). From a transverse perspective, these tesserae are similar to those of the embryo, arranged as a single layer of tightly arranged units separated by very thin strips of uncalcified cartilage and sandwiched between a supratesseral layer and internal uncalcified cartilage (Figures 7C,D, min, uc, SC, IN). From this perspective, the tesserae of *C. milii* most closely resemble the tesserae of the sevengill shark (*Notorynchus cepedianus*) in terms of their arrangement, being very tightly organized and separated by minimal uncalcified cartilage, while also lacking vital chondrocytes; however, they are not comparable in size, being ∼19–57% of the size (Seidel et al., 2016). This is notable, as the Hexanchiformes, to which *N. cepedianus* belongs, are considered one of the most primitive of modern selachian groups (Barnett et al., 2012; Tanaka et al., 2013; da Cunha et al., 2017). However, despite this resemblance, it is likely that this tissue organization does not represent a plesiomorphic trait given the morphology of the skeletal tissues of stem holocephalans such as *Cobelodus* (see section “Mineralised Tissue Development”). It may be that this similarity is an example of evolutionary convergence resulting from common environmental factors. Both *C. milii* and *N. cepedianus* are both known to inhabit lower depths, 200–500 m, respectively (Last and Stevens, 2009; Buglass et al., 2020). This particular skeletal morphology could potentially be beneficial for coping with the conditions of these deep waters. Some osteichthyan fish that also lack swim bladders and inhabit mesopelagic waters (100–1,000 m in depth), have been thought to adapt to the consequences of hydrostatic pressure of these depths through a combination of light “poorly developed” skeletons and “watery” bodies (high water content) to assist with buoyancy and the metabolic costs of locomotion (Blaxter et al., 1971; Blaxter, 1980). Indeed, the scant data on the skeletal biology of deeper-water elasmobranchs (*Somniosus*, *Hexanchus*, *Notorhynchus*) suggests mineralization is greatly reduced, even in adult animals, with tessellation absent or only patchily distributed on skeletal elements (Dean et al., 2015; Seidel et al., 2016; Maisey et al., 2020). Whilst cartilaginous fish are known to control buoyancy through the use of oil-filled livers (Bone and Roberts, 1969; Gleiss et al., 2017), adaptations of the skeletons of these organisms to their environment have never been thoroughly examined. Whether the ostensible convergence in the taxa discussed here is related to common environmental factors could be confirmed by further investigation into the skeletal tissues and ecophysiology of other deep-sea chondrichthyans.

Despite similarities in the early stages of development between elasmobranch and *C. milii* tesserae (e.g., with early growth surrounding vital chondrocytes; Figures 3B, 4E, 5B), the tesserae of *C. milii* differ significantly from those elasmobranchs, and particularly batoids, in terms of size and ultrastructure. The tesserae observed in the stage 36 embryo and adult *C. milii* (Figures 2, 4 and Supplementary Figure 1, min) are much smaller compared to most elasmobranch tesserae that have been examined (Seidel et al., 2016; Figure 10, t), generally being less than 50 μm thick and ranging from 50 to 150 μm in width (Figures 2A, 7C), comparable in size to the ∼100 μm tesserae of the catshark *Scyliorhinus* (Egerbacher et al., 2006; Seidel et al., 2016; Debiais-Thibaud, 2019). Additionally, *C. milii* tesserae display no internal regionalization into the cap and body zones (regions in elasmobranch tesserae delineated by cell shape and collagen type, Figure 10, bz, cz; Kemp and Westrin, 1979; Seidel et al., 2016; Chaumel et al., 2020), with no differences observed between the surfaces closer to the fibrous perichondrium, and surfaces surrounded by hyaline cartilage (e.g., Figures 6A, 7C). *Callorhinchus milii* tesserae also apparently lack the intertesseral joints and mineralized spokes characteristic of elasmobranch tesserae (Figure 10, itj, sp.; Seidel et al., 2016), as well as the Sharpey’s fibers that extend from the perichondrium into the tesserae cap zone in elasmobranchs (e.g., Kemp and Westrin, 1979; Peignoux-Deville et al., 1982; Clement, 1992; Summers, 2000; Seidel et al., 2017a). With respect to growth, the presence of Liesegang lines parallel to tesseral edges (Figures 7C,D, ll) suggests calcification in *C. milii* accretes at the margins of tesserae (Figures 2–5, min, dm, gc) in the same manner as elasmobranch tesserae. Additionally and/or alternatively, *C. milii* tesserae may grow through the development and fusion of new, smaller mineralization foci between existing tesserae (e.g., Figure 6A). Indeed, the irregular and less concentric arrangement of Liesegang lines in *C. milii* tesserae relative to those in elasmobranchs may be indicative of a more multimodal and/or haphazard form of growth, perhaps explaining the varied shape of the observed tesserae (Figure 7).

As noted, chondrocytes appear to be engulfed during mineralization in *C. milii* (Figures 3B,E, 4E) and may be vital in early stages (Figures 4C,D,F, 5, dm, ic). However, more developed tesserae in embryos and adults appear to be acellular (Figures 2, 4A, 6, min), with internal ultrastructures suggesting previously entombed chondrocytes died and underwent micropetrosis, calcifying their lacunae (Figure 7D, ch). Although similar morphological suggestion of micropetrosis has been observed in elasmobranch tesserae (Seidel et al., 2016, 2017b, 2019c), the indication of cell death accompanying mineralization to yield completely acellular tesserae is a major difference to most elasmobranch tesserae (Seidel et al., 2017a; Debiais-Thibaud, 2019; Figure 10). The absence of vital chondrocytes in the tesserae of *C. milii* may have important implications for their maintenance. In batoids, chondrocytes entombed in tesserae (Figure 10) remain vital in uncalcified lacunar spaces and form passages linking adjacent chondrocyte lacunae, similar to the canalaculi found in bone (Dean et al., 2010; Seidel et al., 2016; Chaumel et al., 2020). These chondrocytes and the networks they form are thought to have important functions with regard to maintaining the endoskeleton by communicating information about the mechanical environment in a manner similar to osteocytes in bone (Dean et al., 2010; Seidel et al., 2016; Chaumel et al., 2020). Thus, vital chondrocytes are absent in the mineralized tissues of the adult and the anterior older (anterior) regions of the synarcual, suggesting that these are lost during ontogeny, along with their associated putative mechanosensory networks, and that these functions are either absent or achieved through alternative means.

### Chimaeroid Endoskeleton: Form Across Phylogeny

The limited literature available on chimaeroid skeletal biology offers conflicting descriptions of the endoskeletal mineralization: tessellated calcified cartilage akin to that of elasmobranchs (Hasse, 1879; Seidel et al., 2019b), smooth superficial sheets of continuous calcified cartilage formed from the fusion of tesserae during ontogeny (Lund and Grogan, 1997; Grogan and Lund, 2004; Pradel et al., 2009; Grogan et al., 2015), or a granular texture (*Hydrolagus*, Finarelli and Coates, 2014). Recent histological data from the synarcual of a sub-adult (20 cm) *Hydrolagus* illustrates two forms of mineralized tissue (Debiais-Thibaud, 2019: 116): (1) small (≤50 μm) subperichondral tissues “reminiscent of globular mineralization” at the periphery of the vertebral body and neural arch, that appear to follow a tessellated pattern, and (2) a more irregular form of globular mineralization deep within the vertebral body surrounding the fibrous chordal sheath.

Based on these few recent reported data on chimaeroid mineralization, tesserae in *C. milii* seem to share similarities with those of *Hydrolagus*. In both taxa, mineralization is tessellated and limited to the periphery of structures composed of hyaline cartilage, including the neural arches, basidorsals and basiventrals (vertebral body), though *C. milii* lacks the second deeper layer of globular mineralization (Debiais-Thibaud, 2019; Figure 6.1). The mineralized tissues of *Hydrolagus* also take the form of small, irregular acellular units, lacking clear separation into upper cap and lower body zones (Seidel et al., 2016; Debiais-Thibaud, 2019). Likewise, in *Chimaera*, mineralization more clearly takes the form of tesserae, although differences with respect to the more developed batoid tesserae have been described (Seidel et al., 2019a).

These few recent descriptions of mineralization in modern chimaeroids, including that provided here for *C. millii*, indicate that these taxa neither possess sheets of continuous calcified cartilage, nor a granular texture (Lund and Grogan, 1997; Grogan and Lund, 2004; Pradel et al., 2009; Finarelli and Coates, 2014). Instead they appear to support more historical claims (Hasse, 1879) that these organisms possess tesselated skeletal tissues, though contrary to these sources, these are distinctly different from most elasmobranch tesserae. These discrepant accounts may arise from the tissue arrangements; in taxa such as *C. milii* the tesserae are very tightly arranged, being separated by very thin portions of uncalcified cartilage, which may give the impression that the surface comprises a sheet. The tesserae themselves are covered in a type of fascia (see Materials and Methods, above), which could account for the observations of a granular texture.

Given the quality, available perspectives, and challenging surrounding matrices of many fossil specimens, the identification of useful morphological correlates for identifying tesseral ultrastructures is vital for understanding tesseral evolution and comparing modern and extinct forms. Recently, Maisey et al. (2020) provided a detailed description of the evolution of tesserae in the total group Chondrichthyes, which includes taxa known as acanthodians (Zhu et al., 2013; Coates et al., 2017, 2018; Dearden et al., 2019; Frey et al., 2019). Notably, certain acanthodian taxa, resolved closer to the base of the Chondrichthyes showed “subtesselate calcified cartilage,” where the mineralized layer was broken by fissures, but these did not extend through the layer. More crownward, Maisey et al. (2020) outlined the appearance of various components of the tesserae, for example, the intertesseral joint system in the stem-chondrichthyans *Pucapampella* and *Gladbachus*, and division of the tesserae into cap and body zones in the latter.

As well, the mineralized meshwork visible on jaw cartilages of *Gladbachus* (Coates et al., 2018; Supplementary Figure 2F), echoes a distinct stellate tesseral morphology that has been observed in modern batoid fishes (*Leucoraja erinacea*, *Bathyraja eatonii*, *Urobatis halleri*), termed “trabecular tesserae” by Atake et al. (2019), and which has been shown to be associated with tesserae with a dominant spoke component (i.e., where non-spoke regions of tesserae have been reduced; Jayasankar et al., 2020). This meshwork tissue morphology was also visible when observing the chondral aspect (the “underside”) of the tesseral layer in skates, even in polygonal tesserae (Atake et al., 2019; Figures 1H,I). The association of this meshwork morphology with tesseral spokes and its observation in *Gladbachus* suggests that the stellate/trabecular tesseral morphology and the presence of spokes may be plesiomorphic for the Chondrichthyes and that polygonal tesserae were acquired later in the group (Atake et al., 2019). Although the structural complexity of tesserae is increasingly well-understood (Dean and Summers, 2006; Dean et al., 2009, 2010; Seidel et al., 2016, Seidel et al., 2017, 2019c), it is also increasingly clear that tesserae have had a complex evolutionary history, including the stepwise acquisition of characters.

By comparison, it appears that the Holocephali is characterized by a progressive loss of tesseral features. In a series of stem holocephalans, cartilage mineralization in what are presumed to be adults occurs as small polygonal units that are very similar among disparate taxa (Figure 9). The polygonal shape is more comparable to tesserae in the Elasmobranchii, including the suggested presence of mineralized spokes at tesseral joints in taxa such as *Cobelodus* and potentially *Cladoselache* (Figures 9A–C; Maisey et al., 2020). In contrast, polygonal tesserae are more irregular in shape, and spokes appear absent, in more crownward taxa, including *Edaphodon* (Callorhinchidae), a member of the crown group Holocephali. In these features, the tissues of crownward taxa bear the closest resemblance to those of *C. milii* (Callorhinchidae), however, tesserae in *C. milii* are more irregular in shape and much smaller than the irregular polygonal tesserae of *Edaphodon* (Figure 11C, Supplementary Info Table 1). The apparent loss of characteristics such as spokes and intertesseral joints in the skeletal tissues of crown holocephalans, may be related to their relatively irregular shape and organization. It could potentially be the case that features such as joints and spokes are important in or are a result of the formation of the regular polygonal geometry of tesserae. The presence of shape and structural features in some of the fossil taxa examined that echo those in modern elasmobranch tesserae suggests that substantial changes have occurred in mineralization in living chimaeroids, with a loss of many characteristics of tesserae seen in other chondrichthyans.

## Conclusion

Whilst tessellated cartilage has been suggested to be a shared characteristic of chondrichthyan endoskeletons (e.g., Maisey et al., 2020), the data presented here indicate that this type of mineralization has been significantly modified within the holocephalans. The mineralized layer of the endoskeleton of *Callorhinchus milii* (Family Callorhinchidae) consists of tightly arranged, irregularly shaped tesserae, also present in *Hydrolagus* (Family Chimaeridae). These tesserae in *Callorhinchus* and *Hydrolagus* differ in many respects from most shark and ray tesserae, being smaller and simpler, lacking features such as distinct cap and body zones, mineralized spokes between the tesserae and retention of lacunae housing vital chondrocytes. Nevertheless some similarities in development are present, such as the inter-chondrocyte septa that surround the chondrocytes early in the development of the tesserae, described above in *Callorhinchus* and the ray *Urobatis* (Dean et al., 2009; Seidel et al., 2016). Tesserae in sharks such as *Notorynchus* may also lack some features seen in other elasmobranchs (Debiais-Thibaud, 2019; Figure 6.3; Seidel et al., 2016; Figure 11A). Tesserae in stem group holocephalans, as well as in fossil relatives of *Callorhinchus* such as *Edaphodon*, within the Family Callorhinchidae (Figure 9F), also appear to possess the polygonal shape more characteristic of ray tesserae with these being larger and better developed than the tesserae of adult *Callorhinchus*. Thus it appears that these smaller units may be the characteristic mineralized structure in extant holocephalans, representing a reduction of mineralization occurring separately within the Callorhinchidae and Chimaeridae, and within the Elasmobranchii.

## Data Availability Statement

The raw data supporting the conclusions of this article will be made available by the authors, without undue reservation, to any qualified researcher.

## Ethics Statement

Ethical review and approval from Curtin University was not required for the animal study because specimens of *Callorhinchus milii* were collected near Melbourne several years ago, and CB now works at Curtin University, Perth. She collected these specimens under the following permits: RP1000, RP 1003, and RP1112, with the authorization and direction of the Monash University Animal Ethics Committee (Permit: MAS-ARMI-2010-01). So it’s not necessary for them to approve this particular study, as they approved the initial collection with the appropriate permits.

## Author Contributions

ZJ, CB, and JP conceived this project and contributed data to the project from fossil and extant holocephalans. All authors contributed to interpretation of the data and writing of the manuscript.

## Conflict of Interest

The authors declare that the research was conducted in the absence of any commercial or financial relationships that could be construed as a potential conflict of interest.

## Footnotes

1 https://github.com/AjayLimaye/drishti

2 https://www.fei.com/software/avizo3d/

## Supplementary Material

Supplementary Figure 1 SEM images of mineralization from the synarcual (anterior fused vertebrae) of the second adult *Callorhinchus milii* (Holocephali; Callorhinchidae). **(A)** Tesselated mineralization from a planar perspective; **(B)** close up of mineralization from a planar perspective.

Supplementary Table 1 Holocephali tesserae size (microns).
