# Supplementary figures and images for: Mineralization of the Callorhinchus Vertebral Column (Holocephali; Chondrichthyes)
Source: Front Genet. 2020 Nov 26;11:571694. doi: 10.3389/fgene.2020.571694 (PMC7732695; doi:10.3389/fgene.2020.571694)

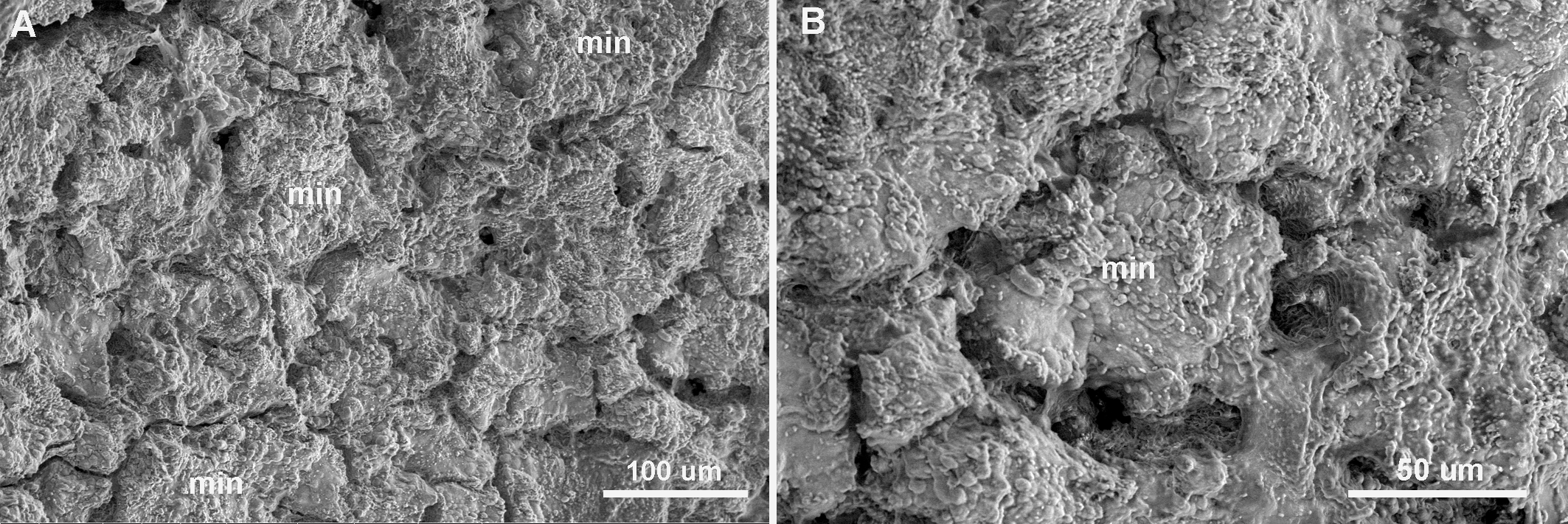

Supplement: Supplementary Figure 1 — SEM images of mineralization from the synarcual (anterior fused vertebrae) of the second adult Callorhinchus milii (Holocephali; Callorhinchidae). (A) Tesselated mineralization from a planar perspective; (B) close up of mineralization from a planar perspective. [file Image_1.TIF]
